# The effect of interictal epileptic discharges and following spindles on motor sequence learning in epilepsy patients

**DOI:** 10.3389/fneur.2022.979333

**Published:** 2022-11-10

**Authors:** Toshiki Okadome, Takahiro Yamaguchi, Takahiko Mukaino, Ayumi Sakata, Katsuya Ogata, Hiroshi Shigeto, Noriko Isobe, Taira Uehara

**Affiliations:** ^1^Department of Neurology, Neurological Institute, Graduate School of Medical Sciences, Kyushu University, Fukuoka, Japan; ^2^Department of Clinical Chemistry and Laboratory Medicine, Kyushu University Hospital, Fukuoka, Japan; ^3^Department of Pharmacy, School of Pharmaceutical Sciences at Fukuoka, International University of Health and Welfare, Okawa, Japan; ^4^Division of Medical Technology, Department of Health Sciences, Graduate School of Medical Sciences, Kyushu University, Fukuoka, Japan; ^5^Department of Neurology, School of Medicine, International University of Health and Welfare Narita Hospital, Narita, Japan

**Keywords:** interictal epileptic discharge, spindle, focal epilepsy, motor sequence learning, non-rapid eye movement sleep

## Abstract

**Purpose:**

Interictal epileptic discharges (IEDs) are known to affect cognitive function in patients with epilepsy, but the mechanism has not been elucidated. Sleep spindles appearing in synchronization with IEDs were recently demonstrated to impair memory consolidation in rat, but this has not been investigated in humans. On the other hand, the increase of sleep spindles at night after learning is positively correlated with amplified learning effects during sleep for motor sequence learning. In this study, we examined the effects of IEDs and IED-coupled spindles on motor sequence learning in patients with epilepsy, and clarified their pathological significance.

**Materials and methods:**

Patients undergoing long-term video-electroencephalography (LT-VEEG) at our hospital from June 2019 to November 2021 and age-matched healthy subjects were recruited. Motor sequence learning consisting of a finger-tapping task was performed before bedtime and the next morning, and the improvement rate of performance was defined as the sleep-dependent learning effect. We searched for factors associated with the changes in learning effect observed between the periods of when antiseizure medications (ASMs) were withdrawn for LT-VEEG and when they were returned to usual doses after LT-VEEG.

**Results:**

Excluding six patients who had epileptic seizures at night after learning, nine patients and 11 healthy subjects were included in the study. In the patient group, there was no significant learning effect when ASMs were withdrawn. The changes in learning effect of the patient group during ASM withdrawal were not correlated with changes in sleep duration or IED density; however, they were significantly negatively correlated with changes in IED-coupled spindle density.

**Conclusion:**

We found that the increase of IED-coupled spindles correlated with the decrease of sleep-dependent learning effects of procedural memory. Pathological IED-coupled sleep spindles could hinder memory consolidation, that is dependent on physiological sleep spindles, resulting in cognitive dysfunction in patients with epilepsy.

## Introduction

Patients with epilepsy demonstrate chronic cognitive dysfunction, which is a major cause of quality of life impairment (1). Epileptic seizures, interictal epileptic discharges (IEDs), antiseizure medications (ASMs), psychiatric symptoms, and brain pathology are thought to be the causes of the cognitive dysfunction (2). IEDs are paroxysmal electroencephalographic findings and occur specifically in patients with epilepsy (3, 4). IEDs reflect the paroxysmal hypersynchronous firings of large neuronal populations, including normative neurons. Replacing the neuronal firings subserving physiological functions with the pathological ones at each IED could lead to transient cognitive dysfunction (5). Actually, there have been many reports about the instantaneous effects of IEDs during cognitive tasks (5–9). Generalized IEDs and IEDs with long duration are known to delay reaction time to stimuli regardless of the type and severity of epilepsy (10, 11). IEDs in the hippocampus and temporal lobe at the encoding and retrieval phase of the verbal memory tasks worsen recall performance in patients with drug-resistant focal epilepsy (12–15). Furthermore, recent studies indicate that IEDs may also have adverse effects on memory processing on a longer time scale, the consolidation of memory (16–18). IEDs during non-rapid eye movement (NREM) sleep particularly cause a decline of recall rate in patients with drug-resistant focal epilepsy (17), suggesting IEDs could deteriorate declarative memory consolidation by replacing neural activity involved in synaptic plasticity during NREM sleep. All of these studies were conducted using declarative memory tasks, and no study investigates the effect of IEDs on long-term memory using procedural memory tasks.

Sleep spindles are neural oscillations observed during NREM sleep with a frequency between 9 and 16 Hz and are associated with various cognitive functions. Especially, they have a central role in memory consolidation, in which synchronous activities across hippocampal sharp-wave ripples, sleep spindles, and cortical slow oscillations enhance hippocampal to cortex information transfer (19). Indeed, reduced coordination between sleep spindles and cortical slow oscillations correlates with impaired memory consolidation in patients with schizophrenia (20) and healthy elderly individuals (21). Among neural activities during NREM sleep, sleep spindles have often been investigated in relation to synaptic plasticity changes, particularly concerning procedural memory. Sleep spindle density increases after procedural memory tasks and the degree of increment correlates with the degree of improvement in performance (22, 23).

Recently, activities in the spindle frequency band following IEDs have been observed during NREM sleep in both rats and humans (24–26). The characteristics of individual IED-coupled spindles, such as duration, amplitude, frequency, and spatial distribution, are no different from physiological ones (24, 26). These findings indicate physiological and IED-coupled spindles share their generation mechanism. Accordingly, it has been hypothesized that IEDs disturb memory consolidation by inducing sleep spindles with inappropriate timing and replacing their physiological counterparts. Actually, these IED-coupled spindles have been reported to impair memory in rats (26) but there have been no reports that directly examine the effects of IED-coupled spindles on cognitive function in humans.

Motor sequence learning, classified as procedural memory, is one of the well-established tasks to study synaptic plasticity during sleep (27, 28). It exhibits a sleep-dependent learning effect, in which the content learned before sleep is enhanced by subsequent sleep without additional training and is attenuated by sleep deprivation, especially in NREM sleep (29, 30). This sleep-dependent learning effect positively correlates with spindle density in NREM sleep after learning (23). Only two studies have investigated sleep-dependent learning effect of patients with epilepsy to our knowledge. One compared the learning effect of epilepsy patients with that of healthy subjects; the former tended to be weaker than the latter, although the difference was not significant (31). The other study examined the impact of epileptic seizures on the learning effect of patients with epilepsy, proposing epileptic seizures themselves could impair learning effect; however, the impact of IEDs was not investigated (32).

The aim of this study is to elucidate the mechanism by which IEDs cause cognitive dysfunction in patients with epilepsy. If the effects of IEDs on cognitive function are clarified in this study, IEDs themselves could be targeted for the treatment of chronic cognitive dysfunction in epileptic patients. We hypothesized that IEDs or IED-coupled spindles during NREM sleep could disrupt sleep-dependent learning effects of motor sequence learning by interfering with physiological neural activity involved in synaptic plasticity. To verify this hypothesis, we conducted motor sequence learning in patients with epilepsy and studied the relationship between results of the overnight electroencephalography (EEG) analysis and sleep-dependent learning effect.

## Materials and methods

The present study was a prospective observational study with minor task intervention in patients with focal epilepsy who were treated at Kyushu University Hospital and age-matched healthy subjects, approved by the Kyushu University Institutional Review Board for Clinical Research (20192003). We attempted to corroborate the existence of sleep-dependent learning disability in patients with epilepsy by comparing their learning effect with healthy controls. Furthermore, we aimed to determine the effect of IEDs and IED-coupled spindles on learning disability by performing the same intervention to the same patient at two time points (Trials 1 and 2) with different IED densities, then analyzing the correlation between the degree of change in learning effect vs. that of IEDs and IED-coupled spindles.

### Subjects selection

In the epilepsy patient group, patients between the ages of 18 and 75 years were included who were diagnosed with focal epilepsy and scheduled to undergo long-term video-electroencephalography (LT-VEEG) with ASM withdrawal at Kyushu University Hospital between June 1, 2019, and December 31, 2021 for clinical necessity. The validity of diagnosis was assessed by at least two expert epileptologists. We excluded patients who had been previously diagnosed with neurological or psychiatric disorders other than epilepsy, had undergone epilepsy surgery, had upper limb motor dysfunction, were professional musicians or typists, or worked at night.

In the healthy group, subjects between ages of 18 and 75 were included who were physically and mentally healthy and were not professional musicians, typists, nor night shift workers.

### Task

The experiment was run in PsychoPy, an open-source experimental-control software package (33). A finger-tapping task was conducted in accordance with previous studies (34–36). Subjects were asked to type on a keyboard a sequence of five numbers from 1 to 4 (e.g., 4-1-3-2-4) with designated fingers of their non-dominant arm (1: index finger, 2: middle finger, 3: ring finger, 4: little finger). We prepared a desktop computer, displayed a digit sequence on the screen, and asked subjects to type the numbers shown on the screen. If they typed correctly, the bar displayed under the number moved to the next number, and if they typed incorrectly, the bar did not move and a warning buzzer sounded. Each block consisted of a 30-s typing period when subjects were instructed to type continuously the numbers as accurately and quickly as possible, followed by a 30-s break period when the subjects were asked to do nothing but stare at the center of the screen. Twelve blocks were performed during the training session before sleep, and three blocks were performed during the retest session the next morning. We prepared two types of digit sequences (4-1-3-2-4 and 2-3-1-4-2) and changed the sequences between Trials 1 and 2 for each subject. The sequence used for each trial was randomly and equally assigned among subjects to avoid bias. The number of digit sequences that were correctly typed for each block was added up, and the average sequence numbers correctly typed during the last three blocks of the training session were compared with those of the retest session (three blocks). The difference between the two sessions was divided by the average of the final three blocks of the training session, multiplied by 100, and expressed as a percentage; this was defined as the sleep-dependent learning effect in this study.

### Overall design

**Figure 1:**
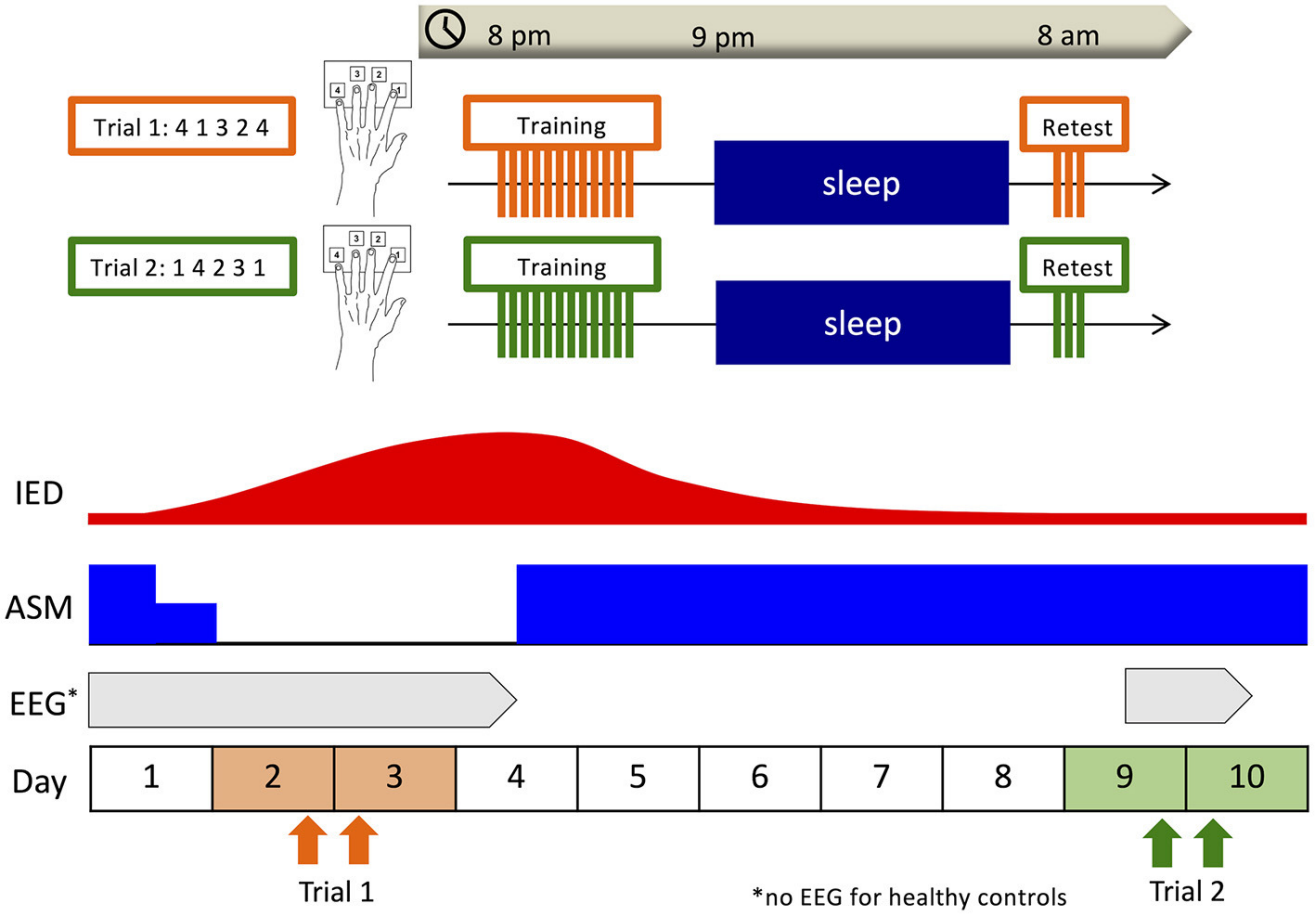
Overall design. Subjects underwent the training session of the finger-tapping task at 8:00 p.m., and the retest session at 8:00 a.m. the next morning. In the patient group, the first trial was conducted on the second night of hospitalization when antiseizure medications (ASMs) were withdrawn for clinical necessity (Trial 1), and the second trial was conducted on the ninth night of hospitalization after ASMs had been returned to usual doses (Trial 2). In the healthy control group, the start date of Trial 1 was set according to the subject's convenience, and Trial 2 was conducted 1 week later. In the patient group, overnight electroencephalography (EEG) was recorded including the nights after each training session as part of long-term video-EEG.

The overall design of this study is shown in Figure 1. Since the measurability of IEDs depends on where the seizure onset zone is located (37), we decided to employ percentage differences for normalization by obtaining IED data of two distinct ASM statuses: the ASM withdrawal period when IEDs are expected to increase and the regular medication period when IEDs are expected to stabilize and diminish. Due to conducting the same learning task twice to a subject within a short timeframe, the potential for the skill and familiarity acquired at the first learning intervention causing a cross-learning effect for the second had to be considered. If there was a difference of learning effect between the two interventions, we could not establish whether the difference was attributed to the cross-learning effect or change in IEDs. Thus, we conducted the same interventions to the healthy group for control. Taking into consideration the minimum time needed to return to a steady state after resuming usual doses of ASMs, the two learning interventions were conducted within a week of each other. This 1-week interval ensured the two interventions could be completed within the same hospital stay. In the healthy group, subjects visited our analysis room in the Kyushu University Hospital at 8:00 p.m., and after a medical interview and brief guidance they underwent the training session of a finger-tapping task. After the training session, the subjects were asked not to practice, recall the task, or use any electronic devices and instruments that required finger tapping, which might interfere with the consolidation of motor sequence learning, and to go to bed as usual. The next morning at 8:00 a.m., they underwent the retest session using the same digit sequence in our analysis room. For the patient group, we visited the patient's room at 8:00 p.m. and the next morning at 8:00 a.m. and administered the same intervention as the healthy subjects. The first trial was conducted on the second night of hospitalization when ASMs were temporarily withdrawn to induce seizures due to clinical necessity as part of LT-VEEG (Trial 1). The second trial was conducted on the ninth night of hospitalization after ASMs were returned to their usual doses (Trial 2). In the healthy control group, the start date of Trial 1 was set according to the subject's convenience, and Trial 2 was conducted 1 week later.

### Data acquisition

In the patient group, an overnight EEG was recorded on the night after each training session as part of LT-VEEG using the Nihon Kohden Neurofax. Nineteen scalp electrodes (Fp1/Fp2/F3/F4/C3/C4/P3/P4/O1/O2/F7/F8/T3/T4/T5/T6/Fz/Cz/Pz) were placed according to the International 10–20 method. The average of C3 and C4 was employed as the system reference for recordings. Referential derivation (unipolar derivation) referring to the electrode placed on the ipsilateral mastoid was used to determine sleep stage and detect spindles, while referential derivation and multiple bipolar derivations were used to detect IEDs. Electrooculogram and electromyogram were recorded simultaneously with EEG. Overnight EEG was recorded in a private hospital ward room specialized for LT-VEEG and soundproofed with adequately controlled temperature. Electrode impedances were measured and dropped below 10 kΩ by skilled clinical neurophysiologists before recording. EEG recordings were constantly checked, and electrode detachment was promptly corrected. All scalp electrodes (Fp1/Fp2/F3/F4/C3/C4/P3/P4/O1/O2/F7/F8/T3/T4/T5/T6/Fz/Pz/Cz) were used to detect IEDs and determine the stage of sleep, and nine electrodes (F3/F4/C3/C4/P3/P4/Fz/Cz/Pz) were used to detect spindles. The sampling frequency was 1 kHz. In line with the 2012 criteria of the American Academy of Sleep Medicine (AASM) (38), two expert electroencephalographers determined sleep stages for every 30-s epoch of EEG recordings from 8:00 p.m. to 8:00 a.m. the next morning. They visually identified spikes, sharp waves, spike-and-slow-wave complexes, sharp-and-slow-wave complexes, polyspike complexes, and polyspike-and-slow-wave complexes during NREM sleep as IEDs using standard amplitude and duration criteria (39). Specifically, a spike or sharp wave was recognized as a transient, clearly distinguished from background activity, with a pointed peak, amplitude larger than 50 μV, and duration between 20 and 70 ms (spike) or 70 and 200 ms (sharp wave). A sequence of two or more spikes was labeled a polyspike complex. They were modified as a spike-and-slow-wave complex, sharp-and-slow-wave complex, or a polyspike-and-slow-wave complex if an associated slow wave separate from background activity followed them. Differences in findings between the two electroencephalographers were reconciled later.

### Spindle autodetection

All analyses of the acquired EEG signals were performed in MATLAB 2018b (The Mathworks Inc., Natick, MA, USA). Spindles were automatically detected in all NREM sleep recordings by the previously adopted algorithm in a study on epilepsy patients and model rats (26). In this algorithm that captures activity above a predefined threshold after normalization, contamination by artificial or pathological high-amplitude signals could precipitate the underestimation of spindles. Therefore, we excluded epochs containing artifacts or visually detected IEDs from the analysis and rejected all outliers before normalization, as described later. The raw data were downsampled to 256 Hz and then notch-filtered at 60 Hz and its multiples to remove the effects of alternating current. The downsampled data were first band-pass filtered from 1 to 50 Hz to extract broadband data, then band-pass filtered from 10 to 20 Hz to extract spindle-band data. After excluding outliers (exceeding the third quartile + 1.5^*^interquartile range), the power values of the amplitudes were extracted using the Hilbert transform and standardized for each electrode by its average value. For discerning spindle shape, we selected continuous activity above 1 standard deviation (SD) with a peak of more than 3 SDs and duration between 0.5 and 2 s as spindle candidates. To exclude false positives stemming from other frequencies, power spectrum analysis was performed on the broadband data within the timeframe of each detected spindle candidate, and those which maximum frequencies were between 11 and 16 Hz were extracted as spindles. These parameters follow a previous study detecting IED-coupled spindles (26). To validate the possible effects of IEDs on spindle detection, we calculated recall value, precision value, and F-score for each patient by comparing the spindles visually identified by a skilled electroencephalographer, independent from the analysis, with those detected by the algorithm (40). Then, we compared these parameters between the patient groups with and without IEDs. This validation was performed on the continuous 1-h sleep records in Trial 1 at C3 when epileptic activities were assumed to be the most influential due to the withdrawal of ASMs. Among the detected spindles, we defined those that began within 1 s after peak of IED as pathological spindles (24, 26). Pathological spindles were detected for each electrode, and the density of pathological spindles was calculated by dividing the number of spindles by the duration of NREM sleep.

### Statistics

All statistical analyses were performed using JMP 14.0 statistical software (SAS Institute, Cary, NC). *P*-value < 0.05 was regarded as statistically significant in all statistical analyses. As epileptic seizures have been reported to impair sleep-dependent learning effects in motor sequence learning, patients who had seizures at the night after training sessions were excluded to focus purely on the effects of IEDs (32). The number of correctly-typed sequences per block was compared between the patient group and control group using a linear mixed model (LMM) for each trial. The number of correctly-typed sequences per block was assigned as the dependent variable; groups (epilepsy and control), blocks (12 training and three retest), and group^*^block interaction as fixed effects; and each subject as a random effect. The means of sleep-dependent learning effect for each group in each trial was compared to zero using the one-sample *t*-test. Moreover, to verify whether there was a difference in sleep-dependent learning effect between Trials 1 and 2 excluding the potential cross-learning effect due to acquired skill and familiarity at Trial 1 affecting learning effect at Trial 2, we compared the means of sleep-dependent learning effect between different groups and trials using a LMM. Sleep-dependent learning effect was set as the dependent variable; the groups (epilepsy and control), trials (Trials 1 and 2), and their interaction as fixed effects; and each subject as a random effect.

In the epilepsy group, we explored possible factors that could have had an association with the change in learning effect between Trials 1 and 2 using simple linear regression analysis with Bonferroni correction. Changes between Trials 1 and 2 in NREM sleep duration, IED density during NREM sleep, all spindle density during NREM sleep, and pathological spindle density during NREM sleep were assessed. The relationship in absolute values, not in changes, between these explanatory variables and the sleep-dependent learning effect was also evaluated separately for each trial. Since pathological spindles assume the presence of IEDs, patients whose EEG did not have discernable IEDs were excluded from regression analyses on IEDs and pathological spindles.

## Results

### Demographics

**Figure 2:**
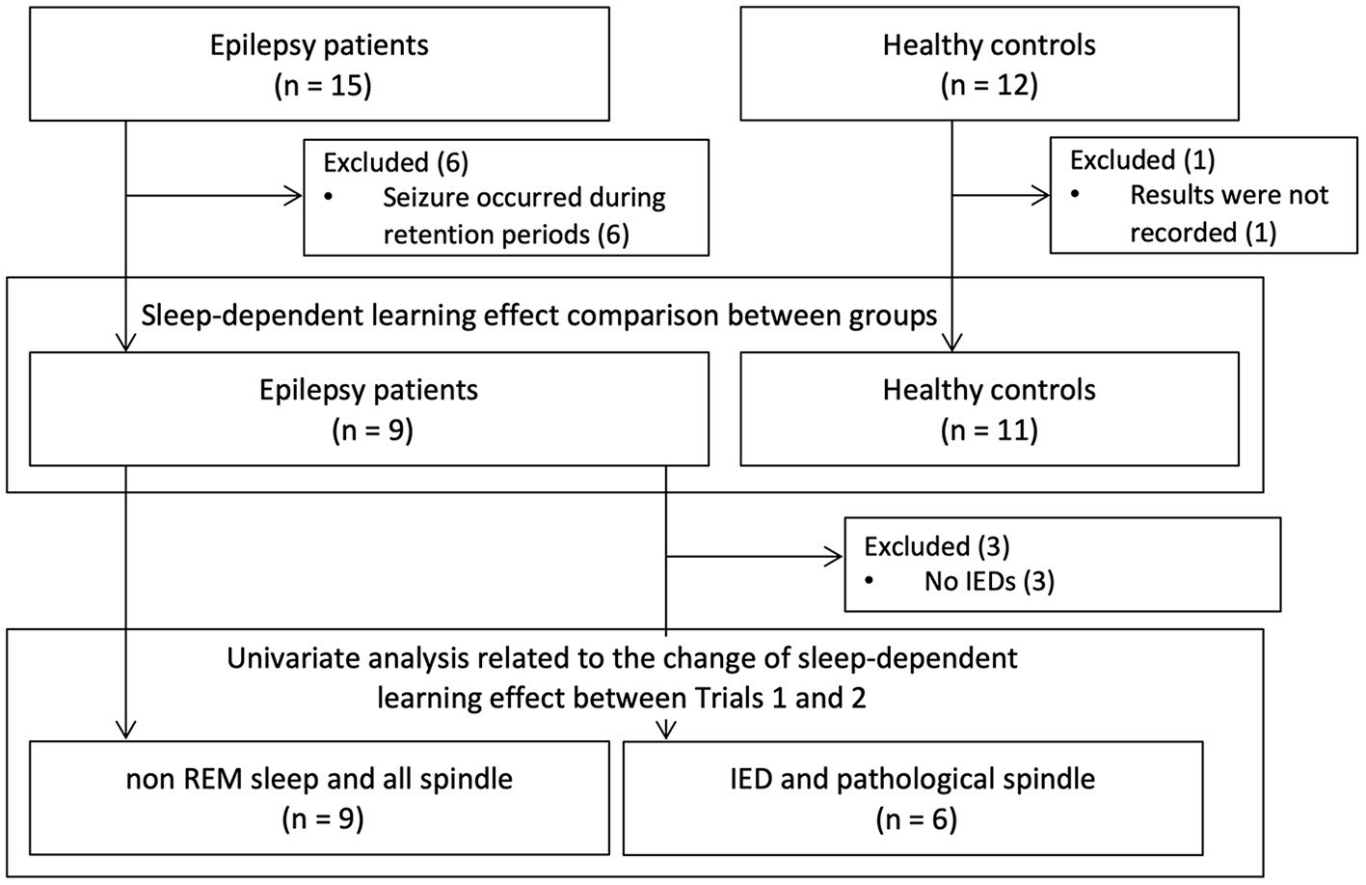
Study population. Fifteen patients with epilepsy and 12 healthy subjects were recruited. Excluding six patients who had epileptic seizures the night after training and one healthy subject whose recordings were defective, we compared sleep-dependent learning effects between nine patients and 11 healthy controls. Then, we exploratively analyzed the factors associated with changes in sleep dependent-learning effect between Trials 1 and 2. When we analyzed about IED and pathological spindle, we excluded three patients who did not have any IEDs. IED, interictal epileptic discharge.

The study population is shown in Figure 2. Fifteen patients with epilepsy and 12 healthy subjects were recruited. Six patients who had epileptic seizures the night after training sessions were excluded. We excluded one healthy subject who had an incomplete study record due to a problem with computer operation. We examined whether there was a difference in sleep-dependent learning effects between the nine epilepsy patients and 11 healthy subjects. We also analyzed the correlation with sleep-dependent learning effects in all the nine patients for NREM sleep duration and all detected spindle density, and in six patients, excluding a further three patients without IEDs (Patients 4, 6, and 9), for IED density and pathological spindle density. The demographics of the nine patients are shown in Supplementary Table 1. Mean age was 28.7 years (SD = 10.6 years) in the patient group and 30.2 years (SD = 10.8 years) in the healthy group, with no significant difference [*t*_(17.5)_, *P* = 0.76]. All subjects in both the patient and healthy groups were right-handed. Age of onset, duration of illness, seizure focus, and ASM varied among patients. We visually detected IEDs in six patients (Patients 1, 2, 3, 5, 7, and 8). The mean IED density among the six patients was 3.3/min (SD = 4.5/min) in Trial 1 and 2.5/min (SD = 3.9/min) in Trial 2. Sleep variables for the patient group are shown in Supplementary Table 2. Upon withdrawal of ASMs, six patients (66.7%) experienced reduction in NREM sleep duration, and all six patients with IEDs exhibited a rise in IED density. When the means of these variables between Trials 1 and 2 were compared by the paired *t*-test, there was a significant difference in IED density [*t*_(5)_ = −3.1, *P* = 0.028], but not in sleep variables (Supplementary Table 2).

### Motor sequence learning and its sleep-dependent learning effect

**Figure 3:**
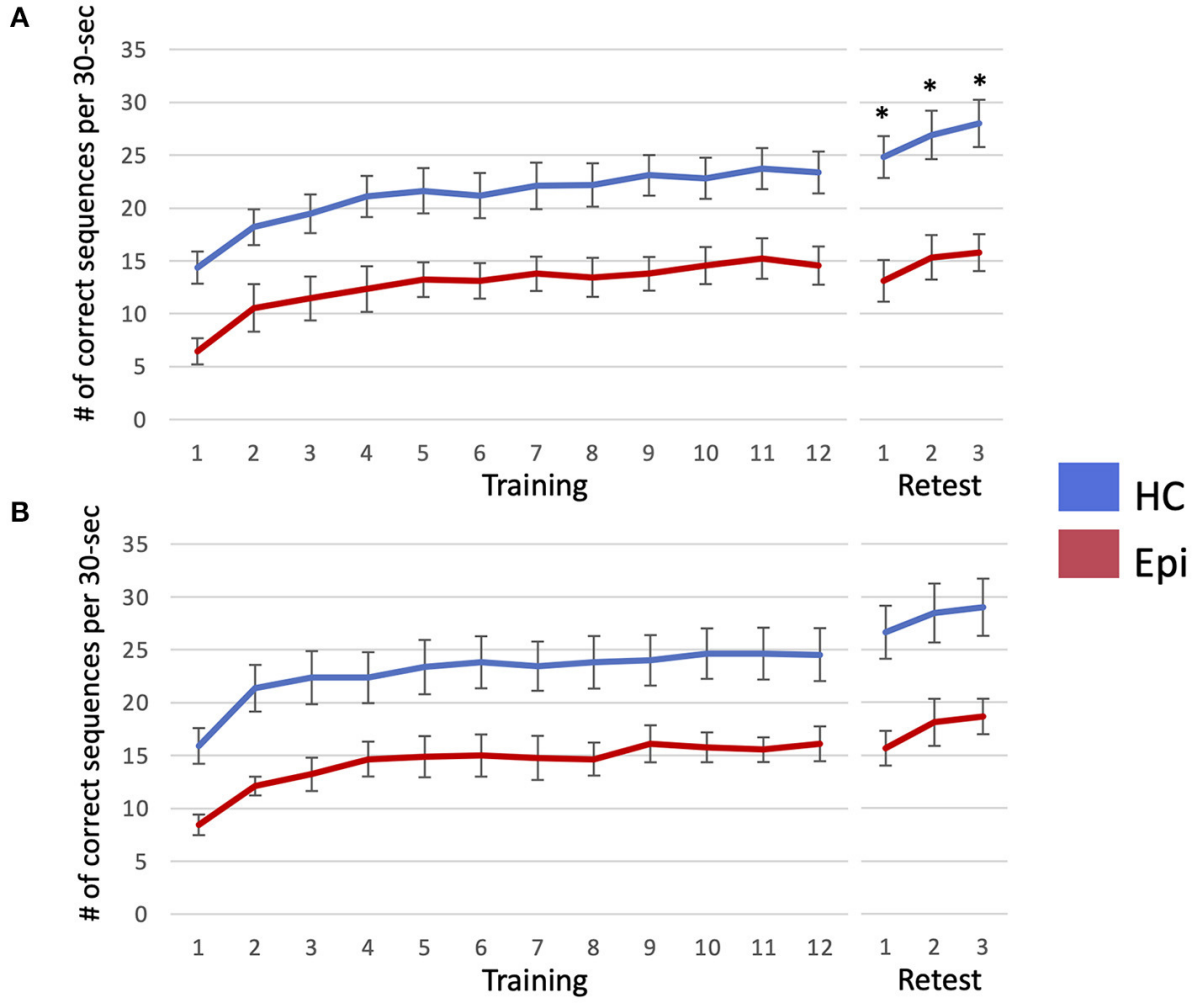
The number of correctly-typed sequences is plotted per each block of Trial 1 **(A)** and Trial 2 **(B)**. Error bar indicates standard error. When the number of correctly-typed sequences were compared between the two groups using a linear mixed model for each trial, that of the healthy control group was significantly higher than that of the epilepsy patient group in all blocks of the retest session in Trial 1, as marked by asterisks. HC, healthy controls; Epi, patients with epilepsy.

**Figure 4:**
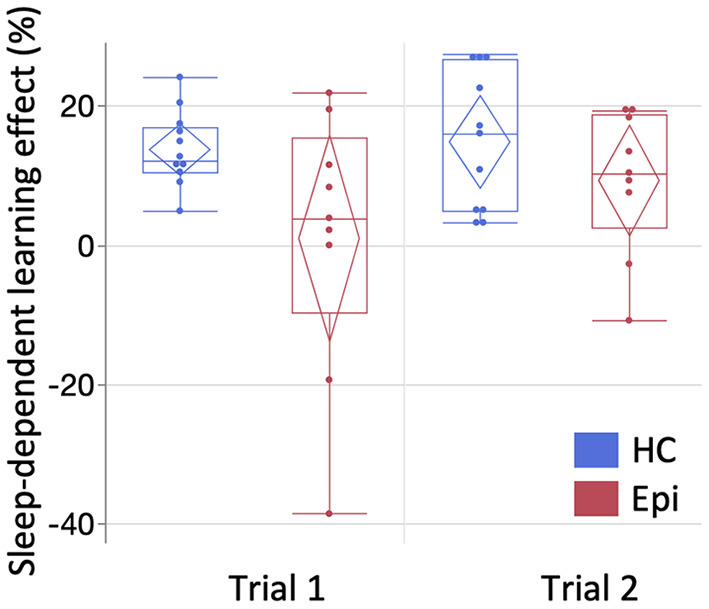
The comparison of sleep-dependent learning effect between groups. The mean sleep-dependent learning effect of the patient group was compared with the healthy group for each trial. Box-and-whisker plot is shown here. Starting from the bottom, each line represents the minimum, lower quartile, median, upper quartile, and maximum. Diamonds indicate the 95% confidence interval of each mean. One-sample *t*-test compared to zero showed significance in Trials 1 [*t*_(10)_ = 8.4, *P* < 0.0001] and 2 [*t*_(10)_ = 5.0, *P* = 0.0006] of the healthy group and in Trial 2 of the epilepsy group [*t*_(8)_ = 2.7, *P* = 0.026], but not in Trial 1 of the epilepsy group [*t*_(8)_ = 0.16, *P* = 0.87]. HC, healthy controls; Epi, epilepsy patients.

In Trial 1, a LMM for number of correctly typed sequences demonstrated the main effects of group [*F*_(1, 18)_ = 11.9, *P* = 0.0028] and block [*F*_(14, 252)_ = 32.2, *P* < 0.0001], and the interaction effect between them to be significant [*F*_(14, 252)_ = 2.3, *P* = 0.0052]. A following *post-hoc* Tukey HSD test clarified that the number of correctly-typed sequences per block in the epilepsy group was significantly lower than in the control group for all blocks of the retest session as shown in Figure 3 [block 1: 13.1 (SD = 5.9) vs. 24.8 (SD = 7.6), *P* = 0.013; block 2: 15.3 (SD = 6.3) vs. 26.9 (SD = 7.4), *P* = 0.015; block 3: 28 (SD = 7.9) vs. 15.8 (SD = 5.2), *P* = 0.0062]. Although a LMM for Trial 2 showed significant main effects in groups [*F*_(1, 18)_ = 9.0, *P* = 0.0076] and blocks [*F*_(14, 252)_ = 38.7, *P* < 0.001], there was no significant interaction between them [*F*_(14, 252)_ = 1.3, *P* = 0.22]. While none of the healthy subjects' performances deteriorated after sleep, two patients (22.2%) in Trial 1 and two patients (22.2%) in Trial 2 had worsened results after sleep. In the healthy group, the mean sleep-dependent learning effect was 13.8% (95%CI = 10.2–17.5%) in Trial 1 and 14.9% (95%CI = 8.24–21.6%) in Trial 2 (Figure 4), and significantly >0 in both Trials 1 [*t*_(10)_ = 8.4, *P* < 0.001] and 2 [*t*_(10)_ = 5.0, *P* = 0.006]. In the patient group, the mean sleep-dependent learning effect was 1.0% (95%CI = −13.7–15.7%) in Trial 1 and 9.3% (95%CI = 1.42–17.3%) in Trial 2, and significantly greater zero in Trial 2 [*t*_(8)_ = 2.7, *P* = 0.026] but not Trial 1 [*t*_(8)_ = 0.16, *P* = 0.87]. A LMM for sleep-dependent learning effect discovered a significant main effect of group [*F*_(1, 18)_ = 4.8, *P* = 0.041], but not of trial [*F*_(1, 18)_ = 2.0, *P* = 0.17], and interaction between group and trial was not significant [*F*_(1, 18)_ = 1.2, *P* = 0.29].

### Pathological spindle

**Figure 5:**
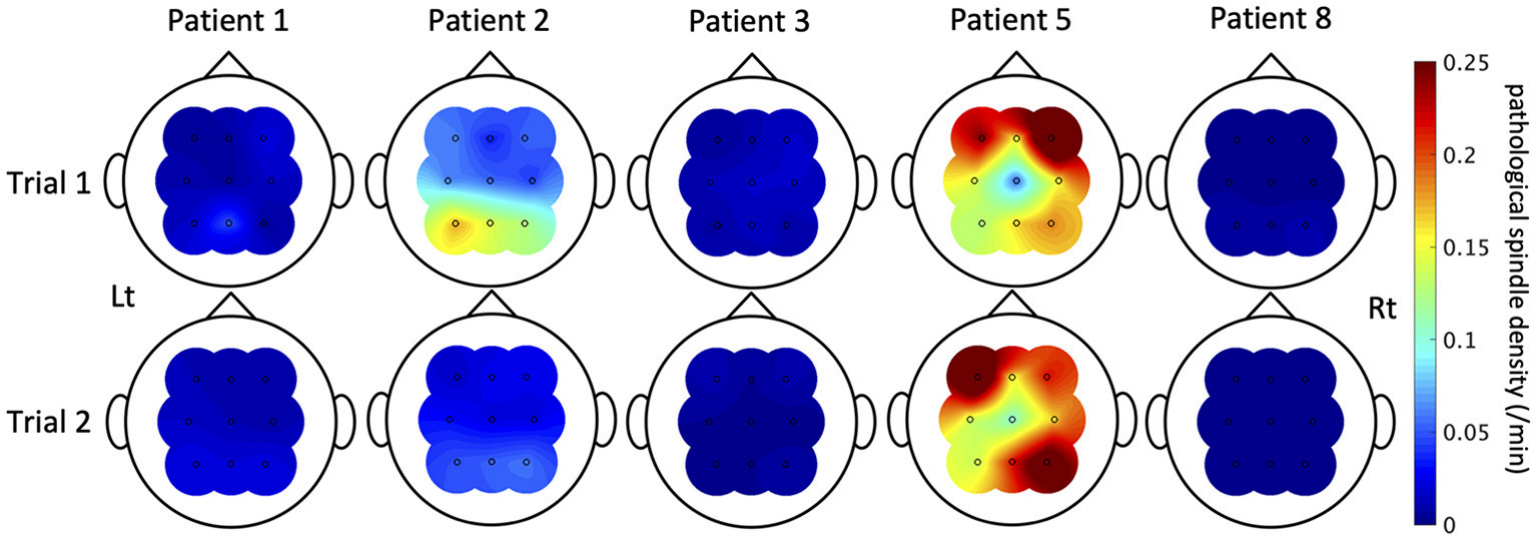
The pathological spindle density distributions. The distributions of interictal epileptic discharge (IED)-coupled spindles are shown here of the five patients in whom IED-coupled spindles were present. The color bar indicates the density of IED-coupled spindles. The nature of fluctuation of IED-coupled spindles between Trials 1 and 2 varied among patients and electrodes.

Spindles were automatically detected from nine electrodes (F3/F4/C3/C4/P3/P4/Fz/Cz/Pz) of each patient. Results validating the algorithm are shown in Supplementary Figure 1. An average value of recall among the nine patients was 0.81 (SD = 0.088), that of precision was 0.88 (SD = 0.074), and that of F-score was 0.85 (SD = 0.073). When we compared the means between patients with IEDs (patients 1, 2, 3, 5, 7, and 8) and those without (patients 4, 6, and 9) by student's *t*-test, there was no significant difference [recall: 0.78 (95% CI = 0.69–0.87) vs. 0.87 (95% CI = 0.73–1.0), *P* = 0.11; precision: 0.89 (95% CI = 0.80–0.97) vs. 0.87 (95% CI = 0.73–1.0), *P* = 0.84; F-score: 0.83 (95% CI = 0.75–0.92) vs. 0.87 (95% CI = 0.74–1.0), *P* = 0.40]. The topography of the mean power and density of all detected spindles including pathological spindles showed symmetric distribution dominant in the centro-parietal region (Supplementary Figure 2), consistent with previous reports (21, 41, 42). The density of all detected spindles had no correlation with age (Supplementary Table 3). Pathological spindles were detected in five of the six patients with IEDs (Patients 1, 2, 3, 5, and 8). Mean pathological spindle density among the five patients was 0.058/min (SD = 0.020/min) in Trial 1 and 0.050/min (SD = 0.017/min) in Trial 2. There was no significant difference between Trials 1 and 2 in the density of all detected spindles and that of pathological spindles for each channel (Supplementary Table 4). In contrast to IEDs, pathological spindles did not necessarily increase uniformly with ASM withdrawal; their pattern of change between Trials 1 and 2 varied among patients and electrodes (Figure 5). Pathological spindles distributed differently among patients and did not overlap with the seizure onset zone.

### The factors associated with sleep-dependent learning effect

**Figure 6:**
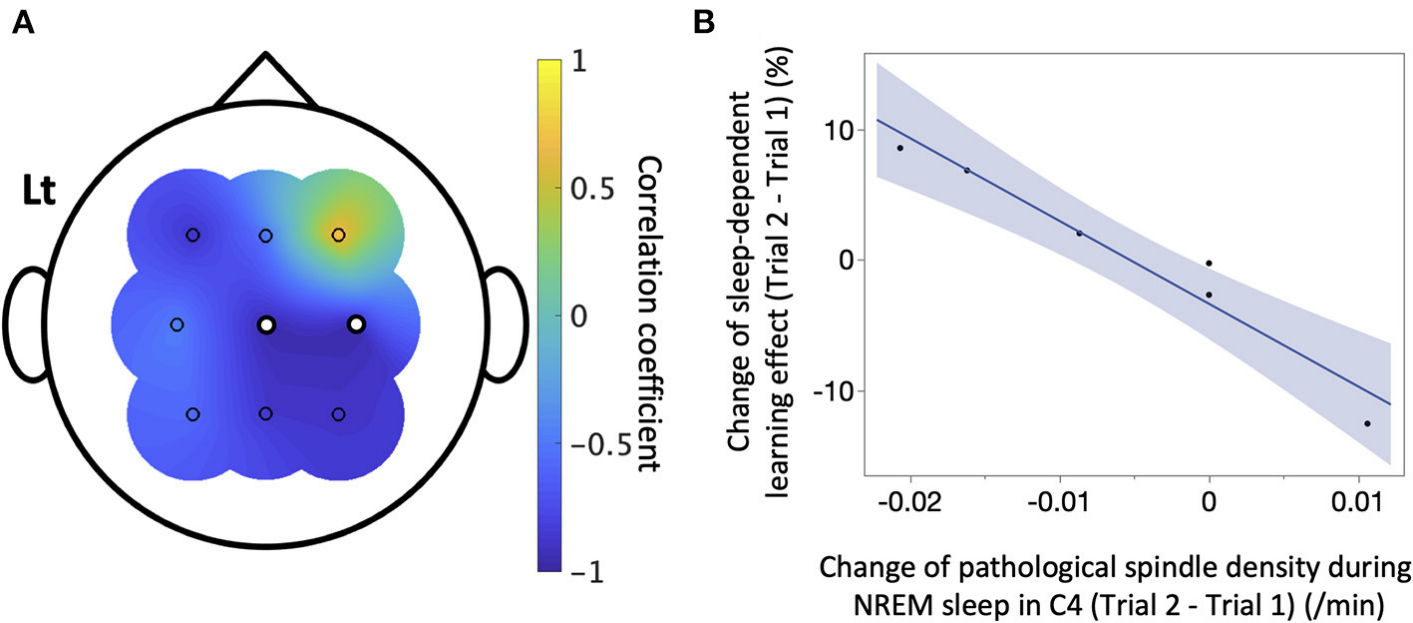
The correlation between sleep-dependent learning effect and pathological spindles. In the six patients with interictal epileptic discharges (IEDs), a simple linear regression analysis was conducted between the change between Trials 1 and 2 of sleep-dependent learning effect and that of IED-coupled spindle density of each electrode. **(A)** The correlation coefficient is shown here. There was a significant negative linear correlation in C4 (β = −634, 95%CI = −858 to −410, *P* = 0.019), marked as white dots. **(B)** A scatter plot of the result for C4 is displayed.

We next explored the factors influencing sleep-dependent learning effects in the patient group (Table 1). Since changes in density of IEDs and pathological spindles showed Poisson distributions, they were converted to the square root. Changes between trials in duration of NREM sleep (β = −0.19, 95% CI = −0.41–0.042, *P* = 0.10), the density of IEDs during NREM sleep (β = 8.1, 95% CI = −1.7–17.9, *P* = 0.082), and the density of all detected spindle during NREM sleep (C4: β = −9.8, 95% CI = −38.1–18.5, *P* = 1.0; the details of other channels are shown in Table 1) were not associated with changes in sleep-dependent learning effect. Conversely, changes in the density of pathological spindles in C4 during NREM sleep had a significant negative correlation with changes in sleep-dependent learning effect (β = −634, 95% CI = −858 to −410, *P* = 0.013) (Figure 6). The results of other channels are shown in Table 1. When focusing on each trial, no significant direct relationship in absolute values was observed between sleep-dependent learning effect and these explanatory variables (Supplementary Table 5).

**Table 1:** Regression analysis with change in sleep-dependent learning effect (Trial 1 – Trial 2)

	**β**	**95% CI**	***P***
**All patients (*****N*** **= 9)**			
Change of NREM sleep duration (Trial 1 – Trial 2)	−0.19	−0.41, 0.042	0.10
Change of all spindle density (Trial 1 – Trial 2)			
F3	4.8	−26.9, 36.4	1.0
F4	8	−29.4, 45.4	1.0
C3	−5.2	−35.6, 25.2	1.0
C4	−9.8	−38.1, 18.5	1.0
P3	−7.4	−27.5, 12.7	1.0
P4	−5.1	−21.0, 10.8	1.0
Fz	8.8	−35.6, 53.2	1.0
Cz	−2.8	−18.0, 12.3	1.0
Pz	−2.9	−27.6, 21.8	1.0
**Patients with IEDs (*****N*** **= 6)**			
Change of IED density during NREM sleep (Trial 1 – Trial 2)	8.1	−1.7, 17.9	0.082
Change of pathological spindle density (Trial 1 – Trial 2)			
F3	−156	−267, −45.5	0.16
F4	101	−36.5, 238.7	1.0
C3	−284	−1,040, 473	1.0
C4	−634	−858, −410	***0.013***
P3	−85	−247, 77.0	1.0
P4	−123	−214, −32.5	0.18
Fz	−881	−2,885, 1,122	1.0
Cz	−276	−382, −171	***0.017***
Pz	−144	−255, −32.8	0.21

Simple regression analysis was conducted of differences between Trials 2 to 1 of sleep-dependent learning effect against of NREM sleep duration and all spindle density for all the nine patients, and IED density and pathological spindle density for the six patients with IEDs. The estimate (β), the 95% CI, and P-value are shown here. P-values were corrected by Bonferroni correction. P < 0.05 was considered significant and shown in bold italic.

## Discussion

The present study is one of the few studies that examine sleep-dependent learning effects of motor sequence learning in patients with epilepsy. We found that there was no significant sleep-dependent learning effect in epilepsy patients when ASM was withdrawn to augment epileptic activity. Furthermore, we found that the increase of IED-coupled spindles, rather than IEDs themselves, significantly correlated with the decrease of sleep-dependent learning effect. This is the first attempt to clarify the pathological significance of IED-coupled spindles in humans.

### Sleep-dependent learning effect

Motor sequence learning is a procedural memory, and whether this kind of memory is impaired in patients with epilepsy has not been well investigated (43). Recently, it has become evident that its sleep-dependent learning effect, especially with regards to spatial component, depends on the hippocampus (44–46); patients with epilepsy with damage to the hippocampus could therefore develop impaired sleep-dependent learning effect. Although the impairment of hippocampus-mediated sleep-dependent memory consolidation has been investigated in the field of declarative memory for patients with focal epilepsy (16), there are still few reports in the field of procedural memory such as motor sequence learning. Deak et al. (31) compared the sleep-dependent learning effects of motor sequence learning between patients with temporal lobe epilepsy and healthy subjects. However, there was no significant difference in learning effect between groups, possibly due to the small number of cases. van Schalkwijk et al. (32) examined the effect of epileptic seizures on motor sequence learning in patients with focal epilepsy and found that sleep-dependent learning effect was reduced in the group with epileptic seizures. There has been no study, however, on IEDs in relation to sleep-dependent learning effect. ASMs could also affect cognitive function in epilepsy patients (47), but it is difficult to align ASMs across patients because ASMs are selected based on individual clinical backgrounds. Therefore, we performed two separate interventions in the same patient, one during ASM withdrawal and the other during usual doses of ASMs, and compared results between the two interventions against controls using a LMM. Nevertheless, the LMM showed no significant interaction between groups and trials in the present study. Low statistical power may be a possible reason for this because when the number of correctly-typed sequences was compared for each trial, the healthy control group showed higher scores than the patient group in the retest session only in Trial 1 (Figure 3A). Furthermore, we found that patients with epilepsy did not display any significant sleep-dependent learning effect only when ASMs were withdrawn. These results suggest that ASM withdrawal and ensuing enhanced IEDs, rather than the disease itself, could affect the learning effect. In this study, we found a significant correlation between changes in learning effect and those in IED-coupled spindle density. However, because IED-coupled spindles can be affected by age, ASM variety (48) and epilepsy type (49), larger sample size experiments controlling for these factors are needed to discuss the causal relationship between IED-coupled spindles and sleep-dependent learning effect.

### Pathological spindles

In an exploratory analysis of factors associated with the changes in sleep-dependent learning effects of motor sequence learning, we found a significant correlation not with the density of IEDs *per se*, but with the density of IED-coupled spindles. This is the first report identifying the pathological significance of IED-coupled spindles in humans. A report that examined the pathological significance of IED-coupled spindles in model rats with temporal lobe epilepsy found an increase in IED-coupled spindles exacerbates memory consolidation impairment (26). The present clinical study affirmed exactly the same results in humans.

Spindles are formed in thalamic reticular nucleus neurons and project to the cortex *via* thalamocortical neurons (50). In NREM sleep, neocortical neurons fire synchronously with the Up state of slow oscillation, which stimulates pacemaker cells in the thalamic reticular nucleus to form a spindle in the thalamus and return it to the neocortex *via* thalamocortical neurons (51–53). These rhythmic and synchronous neural firings consistent with the spindle lead to efficient synaptic excitation of cortical neurons, shaping synaptic plasticity (52). Hippocampal ripples nesting in the slow oscillation-coupled spindles lead to active consolidation of memory *via* hippocampal-thalamic-neocortical synchronization (19). This synchronization among the three regions is essential for consolidating memory, and its artificial desynchronization impairs memory in rats (26, 54). Furthermore, spindles are perceived to have a labeling effect to assist subsequent rescaling by slow-wave sleep, in which the synchrony of spindles with the hippocampus and neocortex determines whether the labeled memory will undergo long-term potentiation or long-term depression (55–60). Although most of these findings relate to declarative memory, findings on procedural memory have been accumulating mainly in the field of schizophrenia in recent years. In patients with schizophrenia, the synchronization between spindles and slow oscillations was impaired and its degree associated with the sleep-dependent learning effect of motor sequence learning (61, 62). Moreover, synchronization among hippocampal ripples, spindles and slow oscillations was impaired in a mouse model of schizophrenia (20). Although there have been no studies directly analyzing the relationship between hippocampal ripples and procedural memory, it has been found that the sleep-dependent learning effect of motor sequence learning is impaired in subjects with lesions in the hippocampus (46). These findings suggest that synchronization among hippocampal ripples, thalamic spindles, and neocortical slow oscillations is important for memory consolidation in procedural memory as well as in declarative memory (63–65).

On the other hand, IEDs consist of sudden hypersynchronous firings initiated by the paroxysmal depolarization shifts of pathological cortical neurons and are followed by inhibitory postsynaptic potentials (3). Since direct electrical stimulation of the localized cortex (54, 66), depolarization of specific neurons by optogenetic techniques (55), as well as transcranial electrical and magnetic stimulation (67) can induce cortical spindles, the synchronous cortical firing by IEDs could also induce spindles in the thalamus *via* thalamocortical neurons (26). However, the erratic nature and timing of spindle formation induced by paroxysmal IEDs would mean they would not be synchronized with neocortical slow oscillations or hippocampal ripples. This could not only interfere with physiological memory consolidation but also promote eliminatory labeling which might debilitate spindle-dependent physiological synaptic plasticity (42, 68–70). Patients in this study who demonstrated an attenuation of sleep-dependent learning effect support this hypothesis. In this study, the harmful effect of IED-coupled spindles was most evident near the motor cortex of the intervening limb (i.e., C4), where the positive impact of physiological spindles on sleep-dependent motor learning is most significant (71–73). This finding might indicate that the replacement of physiological spindles with IED-coupled spindles associated with learning disabilities occur in a region-specific manner, but the confirmation by the further studies with larger sample size are required.

### Limitations

There are several limitations in this study. Firstly, ASMs were not uniform among patients because the baseline ASMs and ASMs to be withdrawn were decided based on clinical necessity. Secondly, the number of cases was small, forcing sample ages and epilepsy types to be heterogeneous, because the study was conducted during the prevalence of COVID-19. The number of patients included in the regression analysis on IED-coupled spindles was further limited to six, leading to low reliability. A study with a larger number of patients with comparable ASMs and ages is desirable. Lastly, because many IEDs and spindles could only be detected by intracranial recordings, we might have underestimated IEDs and IED-coupled spindles. In the future, we hope to perform similar interventions on patients with intracranial electrodes.

## Conclusion

We found that patients with epilepsy showed no significant sleep-dependent learning effect of procedural memory during the ASM withdrawal period. Furthermore, we identified that the decrease of sleep-dependent learning effect significantly correlated with the increase of IED-coupled spindles and not the IEDs themselves. This is the first attempt to elucidate the pathological significance of IED-coupled spindles in humans.

## Data availability statement

The original contributions presented in the study are included in the article/Supplementary material, further inquiries can be directed to the corresponding author.

## Ethics statement

The studies involving human participants were reviewed and approved by the Kyushu University Institutional Review Board for Clinical Research (20192003). The patients/participants provided their written informed consent to participate in this study.

## Author contributions

TO and TU have contributed to the conception and design of the study. TO, TY, TM, AS, KO, HS, and TU have contributed to the acquisition and analysis of data. TO, HS, NI, and TU have contributed to the drafting and revising the manuscript. All authors contributed to the article and approved the submitted version.

## Funding

HS and TU was supported by a grant from JSPS KAKENHI (Grant No. 19K07964). NI was supported by a grant from JSPS KAKENHI (Grant No. 21K07464).

## Conflict of interest

The authors declare that the research was conducted in the absence of any commercial or financial relationships that could be construed as a potential conflict of interest.

## Publisher's note

All claims expressed in this article are solely those of the authors and do not necessarily represent those of their affiliated organizations, or those of the publisher, the editors and the reviewers. Any product that may be evaluated in this article, or claim that may be made by its manufacturer, is not guaranteed or endorsed by the publisher.

## Abbreviations

ASM: antiseizure medication
IED: interictal epileptic discharge
LT-VEEG: log-term video-encephalography
NREM: non-rapid eye movement.
