# Supplementary material for: The effect of interictal epileptic discharges and following spindles on motor sequence learning in epilepsy patients
Source: Front Neurol. 2022 Nov 10;13:979333. doi: 10.3389/fneur.2022.979333 (PMC9686303; doi:10.3389/fneur.2022.979333)
Supplement: Supplementary file 1 [file Data_Sheet_1.docx]

Supplementary Material

**Supplementary Table 1. Patient demographics**

| **Patient** | 1 | 2 | 3 | 4 | 5 | 6 | 7 | 8 | 9 |
| --- | --- | --- | --- | --- | --- | --- | --- | --- | --- |
| **Clinical demographics** | | | | | | | | | |
| Age at intervention (years old) | 41 | 50 | 19 | 31 | 29 | 21 | 18 | 21 | 23 |
| Gender | Male | Male | Male | Male | Male | Female | Male | Male | Female |
| Dominant hand | Rt | Rt | Rt | Rt | Rt | Rt | Rt | Rt | Rt |
| Age at onset (years old) | 5 | 46 | 24 | 26 | 15 | 0 | 14 | 9 | 13 |
| Disease duration (years) | 37 | 5 | 6 | 6 | 15 | 21 | 5 | 13 | 11 |
| Laterality | Lt | Rt | Lt | Lt | Lt | Rt | Rt | Lt | Rt |
| Speculated epileptogenic region | Frontal | Temporal | Frontal | Temporal | Temporal | Temporal | Occipital | Frontal | Frontal |
| Baseline ASM | CBZ, CLB, PB, PHT | LCM, LEV, VPA | LCM | VPA | LEV, PER, ZNS | LCM, LEV | LTG | CBZ, CLB, LTG | LTG, CZP |
| ASM withdrawn at Trial 1 | CBZ, CLB, PHT | LEV, LCM | LCM | VPA | LEV | LCM | LTG | CBZ, LTG | LTG, CZP |
| **Motor sequence learning** | | | | | | | | | |
| Trial 1 | | | | | | | | | |
| Mean cSq # of the last 3 trainings | 8 | 10.3 | 17.3 | 15.3 | 10.7 | 25.7 | 13.7 | 13 | 19 |
| Mean cSq # of retest | 8.7 | 8.3 | 19.3 | 15.7 | 13 | 26.7 | 16.3 | 13 | 11.7 |
| Sleep-dependent learning effect (%) | 8.3 | -19.3 | 11.5 | 2.2 | 21.9 | 3.9 | 19.5 | 0 | -38.6 |
| Trial 2 | | | | | | | | | |
| Mean cSq # of the last 3 trainings | 9.7 | 12.3 | 16.3 | 17.3 | 14.3 | 22 | 17.3 | 12.3 | 20.7 |
| Mean cSq # of retest | 10.7 | 11 | 19.3 | 19.7 | 15.7 | 23.7 | 20.7 | 12 | 24.7 |
| Sleep-dependent learning effect (%) | 10.3 | -10.8 | 18.4 | 13.5 | 9.3 | 7.6 | 19.2 | -2.7 | 19.4 |
| **Sleep records** | | | | | | | | | |
| NREM sleep duration (min) | | | | | | | | | |
| Trial 1 | 264 | 365 | 283 | 469 | 318 | 395 | 231 | 357 | 444 |
| Trial 2 | 323 | 405 | 334 | 442 | 400 | 396 | 394 | 350 | 324 |
| IED density in NREM sleep (/min) | | | | | | | | | |
| Trial 1 | 0.6 | 5.8 | 0.4 | NE | 11.4 | NE | 0.3 | 1.3 | NE |
| Trial 2 | 0.1 | 5 | 0.1 | NE | 9.4 | NE | 0.008 | 0.3 | NE |
| Pathological spindle density in NREM sleep (/min) | | | | | | | | | |
| Trial 1 | 0.015 | 0.083 | 0.012 | NE | 0.18 | NE | NE | 0.003 | NE |
| Trial 2 | 0.015 | 0.033 | 0.004 | NE | 0.19 | NE | NE | 0.002 | NE |
| Rt = right; Lt = left; ASM = antiseizure medication; cSq = correctly-typed sequence; CBZ = carbamazepine; CLB = clobazam; CZP = clonazepam; PB = phenobarbital; PER = perampanel; PHT = phenytoin; LCM = lacosamide; LEV = levetiracetam; LTG = lamotrigine; VPA = valproate; NREM = non-rapid eye movement; IED = interictal epileptic discharge; NE = not extracted. | | | | | | | | | |

**Supplementary Table 2. Sleep variables and IED demographics**

|  |  | **Trial 1** | **Trial 2** | **P** |
| --- | --- | --- | --- | --- |
| Sleep variables (N = 9) | | | | |
| Total bed time (min) | mean (SD) | 511 (58.5) | 471.4 (80.4) | 0.32 |
| Total sleep time (min) | mean (SD) | 390 (96.6) | 425.7 (55.2) | 0.31 |
| N1 (min) | mean (SD) | 66.7 (26.5) | 66.9 (16.9) | 0.98 |
| N2 (min) | mean (SD) | 205.1 (63.2) | 213.1 (29.4) | 0.66 |
| N3 (min) | mean (SD) | 75.6 (29.1) | 92 (25.7) | 0.084 |
| REM (min) | mean (SD) | 41.9 (25.4) | 53 (21.6) | 0.16 |
| Sleep efficiency index (%) | mean (SD) | 0.76 (0.15) | 0.88 (0.074) | 0.066 |
| WASO (min) | mean (SD) | 110.4 (76.5) | 59.9 (36.4) | 0.12 |
| # of arousals | mean (SD) | 20.6 (7.5) | 21.7 (4.7) | 0.51 |
| IED (N = 6) | | | | |
| density (/min) | mean (SD) | 3.3 (4.5) | 2.5 (3.9) | ***0.031*** |
| The means and SDs of each sleep variable from all nine patients and those of IED density from the six patients who had IEDs are shown here. Differences between Trials 1 and 2 were compared with paired t-test. P < 0.05 was considered significant and is shown in bold italic. WASO = wake after sleep onset. | | | | |

**Supplementary Table 3. Regression analysis with age for each trial**

|  | **Trial 1** | | |  | **Trial 2** | | |
| --- | --- | --- | --- | --- | --- | --- | --- |
|  | β | 95% CI | P |  | β | 95% CI | P |
| sleep-dependent learning effect | -0.41 | -1.9, 1.2 | 0.51 |  | -0.56 | -1.3, 0.15 | 0.10 |
| all spindle density | | | | | | | |
| F3 | 0.13 | -0.58, 0.73 | 1.0 |  | 0.08 | -0.61, 0.71 | 1.0 |
| F4 | 0.079 | -0.62, 0.70 | 1.0 |  | 0.05 | -0.63, 0.69 | 1.0 |
| C3 | 0.28 | -0.47, 0.80 | 1.0 |  | 0.19 | -0.54, 0.76 | 1.0 |
| C4 | 0.17 | -0.56, 0.75 | 1.0 |  | 0.15 | -0.57, 0.74 | 1.0 |
| P3 | 0.32 | -0.44, 0.81 | 1.0 |  | 0.28 | -0.47, 0.80 | 1.0 |
| P4 | 0.16 | -0.56, 0.75 | 1.0 |  | 0.21 | -0.53, 0.77 | 1.0 |
| Fz | 0.088 | -0.61, 0.71 | 1.0 |  | 0.07 | -0.62, 0.70 | 1.0 |
| Cz | -0.034 | -0.68, 0.64 | 1.0 |  | 0.24 | -0.51, 0.78 | 1.0 |
| Pz | 0.39 | -0.37, 0.84 | 1.0 |  | 0.24 | -0.51, 0.78 | 1.0 |
| A simple regression analysis between age and each factor was conducted for each trial. The estimate (β), 95% CI, and P value are listed. P values have been corrected by Bonferroni correction. P < 0.05 was considered significant. There was no significant linear correlation between age and each factor. | | | | | | | |

**Supplementary Table 4. Changes in spindle density**

|  |  | **all detected spindles (N = 9)** | | |  | **pathological spindles (N = 5)** | | |
| --- | --- | --- | --- | --- | --- | --- | --- | --- |
|  |  | Trial1 | Trial2 | P |  | Trial1 | Trial2 | P |
| F3 (/min) | mean (SD) | 2.2 (1.6) | 2.2 (1.7) | 0.79 |  | 0.063 (0.11) | 0.075 (0.14) | 0.61 |
| F4 (/min) | mean (SD) | 2.1 (1.6) | 2.1 (1.7) | 0.93 |  | 0.089 (0.15) | 0.053 (0.092) | 0.24 |
| C3 (/min) | mean (SD) | 2.3 (1.7) | 2.2 (1.5) | 0.72 |  | 0.046 (0.058) | 0.037 (0.057) | 0.2 |
| C4 (/min) | mean (SD) | 2.1 (1.5) | 2.2 (1.5) | 0.72 |  | 0.046 (0.063) | 0.039 (0.071) | 0.28 |
| P3 (/min) | mean (SD) | 2.9 (1.6) | 3.2 (1.8) | 0.43 |  | 0.067 (0.080) | 0.042 (0.057) | 0.39 |
| P4 (/min) | mean (SD) | 2.6 (1.5) | 3.1 (1.6) | 0.22 |  | 0.067 (0.083) | 0.075 (0.12) | 0.78 |
| Fz (/min) | mean (SD) | 1.9 (1.5) | 1.9 (1.5) | 0.57 |  | 0.040 (0.063) | 0.038 (0.063) | 0.48 |
| Cz (/min) | mean (SD) | 1.9 (1.5) | 2.2 (1.8) | 0.49 |  | 0.026 (0.026) | 0.027 (0.042) | 0.91 |
| Pz (/min) | mean (SD) | 3.3 (1.8) | 3.5 (1.7) | 0.43 |  | 0.076 (0.073) | 0.059 (0.091) | 0.5 |
| For each channel, the density of all detected spindles from nine patients and that of pathological spindles from the five patients who exhibited them are shown here. Differences between Trials 1 and 2 were compared with the paired t-test. P < 0.05 was considered significant. There were no significant differences between Trials 1 and 2 for both all detected spindles and pathological spindles. | | | | | | | | |

**Supplementary Table 5. Regression analysis with sleep-dependent learning effect for each trial**

|  | **Trial 1** | | |  | **Trial 2** | | |
| --- | --- | --- | --- | --- | --- | --- | --- |
|  | β | 95% CI | P |  | β | 95% CI | P |
| All patients (N = 9) | | | | | | | |
| NREM sleep duration | -0.13 | -0.34, 0.091 | 0.21 |  | -0.068 | -0.38, 0.25 | 0.63 |
| all spindle density | | | | | | | |
| F3 | 0.33 | -10.5, 11.2 | 1.0 |  | 3.2 | -1.6, 7.9 | 1.0 |
| F4 | 1.3 | -9.3, 11.8 | 1.0 |  | 3.1 | -1.9, 8.0 | 1.0 |
| C3 | -0.0041 | -10.3, 10.3 | 1.0 |  | 3.6 | -1.7, 8.9 | 1.0 |
| C4 | 0.19 | -10.9, 11.3 | 1.0 |  | 3.2 | -2.1, 8.6 | 1.0 |
| P3 | -2.4 | -13.2, 8.4 | 1.0 |  | 2.8 | -2.2, 7.9 | 1.0 |
| P4 | -2.6 | -13.7, 8.4 | 1.0 |  | 3.4 | -1.4, 8.3 | 1.0 |
| Fz | 0.057 | -11.7, 11.8 | 1.0 |  | 3.7 | -1.6, 9.0 | 1.0 |
| Cz | -2.0 | -13.0, 9.1 | 1.0 |  | 2.7 | -2.8, 8.2 | 1.0 |
| Pz | -1.8 | -11.1, 7.5 | 1.0 |  | 3.1 | -1.7, 7.9 | 1.0 |
|  |  |  |  |  |  |  |  |
| Patients with IEDs (N = 6) | | | | | | | |
| IED density during NREM sleep | 0.15 | -4.5, 4.8 | 0.93 |  | -0.95 | -4.9, 3.0 | 0.54 |
| pathological spindle density | | | | | | | |
| F3 | 43.4 | -162, 250 | 1.0 |  | 5.8 | -118, 130 | 1.0 |
| F4 | 41.4 | -99.1, 182 | 1.0 |  | 3.0 | -191, 197 | 1.0 |
| C3 | 35.9 | -338, 411 | 1.0 |  | -14 | -326, 298 | 1.0 |
| C4 | 73.9 | -267, 415 | 1.0 |  | -1.5 | -253, 250 | 1.0 |
| P3 | -94.5 | -336, 148 | 1.0 |  | -3.45 | -379, 372 | 1.0 |
| P4 | -19.1 | -282, 244 | 1.0 |  | -5.4 | -154, 144 | 1.0 |
| Fz | 85.5 | -256, 427 | 1.0 |  | -10 | -293, 273 | 1.0 |
| Cz | -161 | -949, 626 | 1.0 |  | -24 | -447, 398 | 1.0 |
| Pz | -44.9 | -328, 238 | 1.0 |  | -12 | -207, 182 | 1.0 |
| For each trial, simple regression analysis was conducted between sleep-dependent learning effect against NREM sleep duration and all spindle density for all the nine patients, and IED density and pathological spindle density for the six patients with IEDs. The estimate (β), the 95% CI, and P value for each explanatory variable are shown here. P values were corrected by Bonferroni correction. P < 0.05 was considered significant. | | | | | | | |


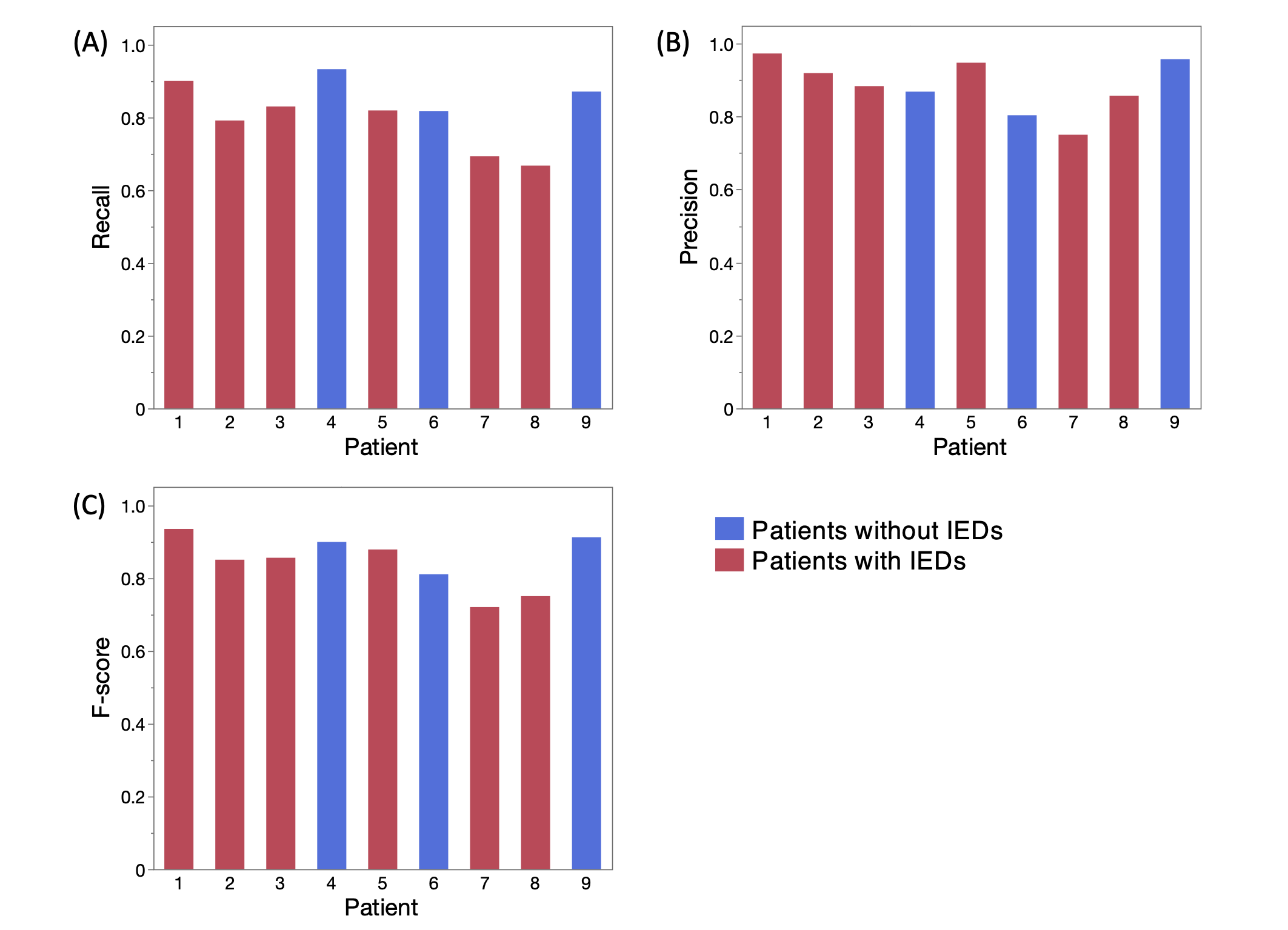


**Supplementary Figure 1. Validation results of spindle autodetection algorithm**

Recall values, precision values, and F-scores of automatically detected spindles are shown here for each patient. These scores were calculated by comparing the spindles visually identified by a skilled electroencephalographer, independent from the analysis, with those detected by autodetection algorithm in one-hour sleep records for Trial 1 at C3.


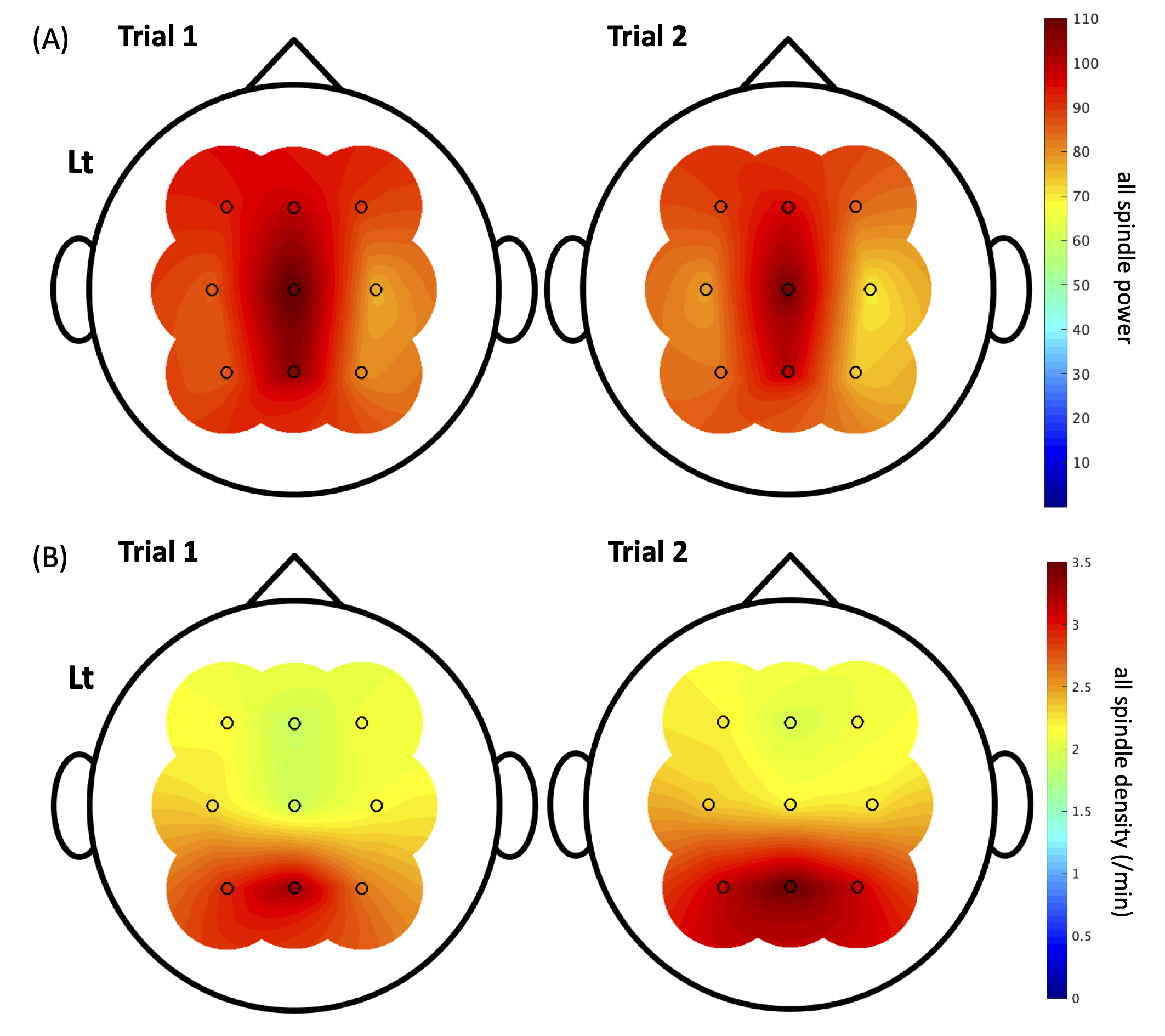


**Supplementary Figure 2. The topography of all detected spindles**

The average power (A) and density (B) topography of all detected spindles among nine patients is shown here for each trial. A symmetrical distribution dominant in the centro-parietal region is seen in both Trials 1 and 2.


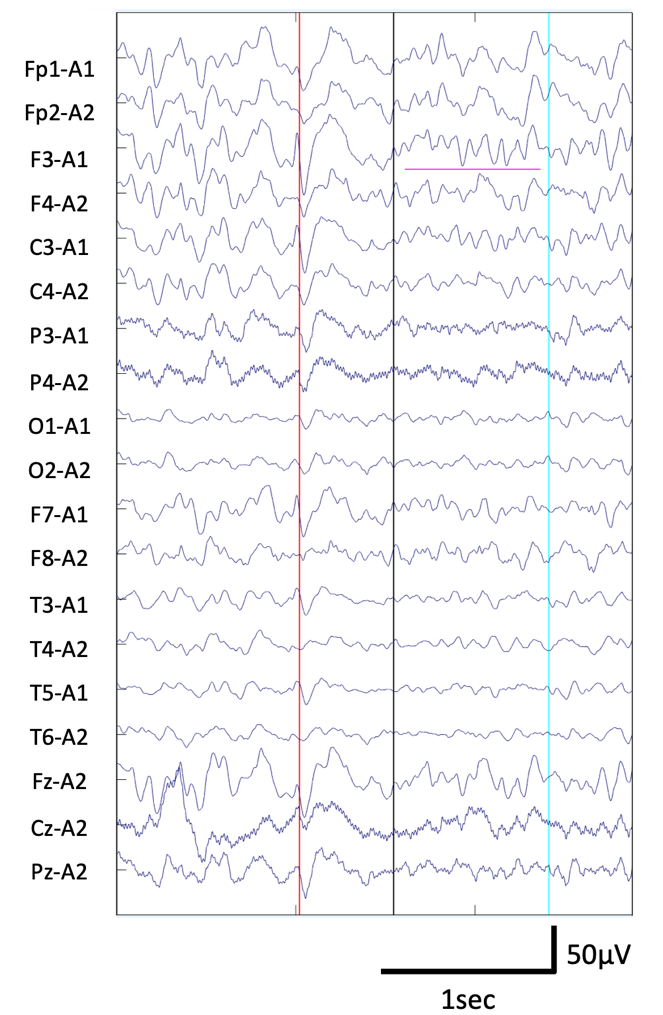


**Supplementary Figure 3. Example of an IED-coupled spindle**

An IED-coupled spindle of patient 2 is shown here. EEG is recorded using ipsilateral mastoid electrodes as reference. The magenta underline identifies an IED-coupled spindle in F3. The red line indicates the peak of the IED, while black and cyan lines indicate the start and end of the IED-coupled spindle respectively.
